# Dissipation and Residue of Chloroisobromine Cyanuric Acid in Ginger and Pepper and Its Dietary Intake Risk Assessment

**DOI:** 10.3390/foods13203247

**Published:** 2024-10-12

**Authors:** Yadong Hao, Yingxuan Li, Jue Wang, Sen Pang, Shuang Li

**Affiliations:** 1 Beijing Haidian District Food and Drug Safety Monitoring Center, Beijing 100094, China; 695870046gg@gmail.com; 2 College of Land Science and Technology, China Agricultural University, Beijing 100193, China; b20213210993@cau.edu.cn; 3 College of Science, China Agricultural University, Beijing 100193, China; wangjue@chemeva.com (J.W.); pangsen7812@cau.edu.cn (S.P.); 4 College of Information and Electrical Engineering, China Agricultural University, Beijing 100193, China

**Keywords:** chloroisobromine cyanuric acid, cyanuric acid, pepper, ginger, residue, dissipation, dietary risk

## Abstract

Chloroisobromine cyanuric acid is a highly effective broad-spectrum systemic fungicide for disease control in agricultural crops. In this study, the degradation, residue levels, and potential dietary risks associated with the chloroisobromine cyanuric acid residues in two widely consumed crops, pepper and ginger, were evaluated through supervised field experiments conducted at 12 sites for pepper and 4 sites for ginger in China in 2021. A QuEChERS-LC-MS/MS method was established for quantifying cyanuric acid (CYA) in both crops. The method achieved a limit of quantification (LOQ) of 0.02 mg kg^−1^ for ginger and 0.05 mg kg^−1^ for pepper, with recovery rates ranging from 91% to 96% for ginger and 84% to 89% for pepper and the relative standard deviation of 2.9% to 11.2% for ginger and 2.8%~12.9% for pepper, respectively. The results indicated that CYA had half-lives ranging from 3.1 to 8.2 days in pepper, and the terminal CYA residues at harvest were all lower than 5 mg kg^−1^, the maximum residue limit established in China. Furthermore, the chronic dietary risk exposure to chloroisobromine cyanuric acid in ginger and pepper, harvested at a pre-harvest interval of three days and at the normal harvesting time after the application of 50% chloroisobromine cyanuric acid soluble powder (SP), was 37.96%, which was much lower than 100%, indicating no significant health risks to the Chinese population. This study provides basic data for establishing the MRLs and serving as a reference for developing the analytical method applicable to CYA residues in different plant matrices.

## 1. Introduction

Chloroisobromine cyanuric acid stands as an innovative, highly efficient, and broad-spectrum systemic fungicide that demonstrates remarkable potency against a wide array of bacteria, algae, fungi, and pathogens. Chloroisobromine cyanuric acid is not only widely used in water supply companies, swimming pools, and medical facilities, but also is increasingly used in the control of crop diseases. As an oxidizing bactericide, chloroisobromine cyanuric acid operates by gradually releasing hypochlorous acid and hypobromous acid onto the crop’s surface. These two acids penetrate the cell membrane of pathogenic organisms through diffusion and interact with their internal protoplasm to disrupt their physiological functions, ultimately leading to the death of these pathogens. It has been reported that chloroisobromine cyanuric acid has good control of tobacco mosaic virus disease, cabbage soft rot disease, cucumber downy mildew disease, bacterial leaf streak, bacterial blight of rice, and so on [1,2,3]. According to the query data of the China Pesticide Information Network, currently, as a pesticide ingredient, chloroisobromine cyanuric acid has been registered as a pesticide ingredient in cabbage, tobacco, rice, tomato, and cucumber for the control of a wide range of diseases in China. However, there is only one registered product for pepper and none yet for ginger [4].

The residue of chloroisobromine cyanuric acid is identified as cyanuric acid (CYA) according to its residue definition outlined in “The List of Residues to be Tested in Pesticide Registration Residue Test and Plant-Derived Food Dietary Risk Assessment”, issued by the Ministry of Agriculture and Rural Affairs of the People’s Republic of China [5]. The official maximum residue level (MRL) established by the Chinese government was 5 mg kg^−1^ for chloroisobromine cyanuric acid in pepper, with no MRL given for ginger in China, and the acceptable daily intake (ADI) value was listed as 0.007 mg kg^−1^ of body weight [6]. Therefore, there is a need to test ginger for chloroisobromine cyanuric acid pesticide residues and to assess its dietary intake risk. 

Currently, there are many reports on the detection of CYA in water, dairy, feed, and alimentary products as a metabolite of melamine [7,8,9,10,11,12]. However, most of these methods are not suitable for determining the residues in plants. The reason for the inapplicability of these methods may be that they lack the sensitivity or selectivity to determine the low levels of CYA typically found in complex plant matrices such as ginger and pepper. There are relatively few studies on detection methods and residue data for residues of chloroisobromine cyanuric acid in crops. Wang et al. established an analytical method for tobacco and soil using HPLC after extraction with acetonitrile and purification with trichloromethane and petroleum ether, but the method used more organic solutions, and was complicated and time-consuming to operate [13]. Similarly, Li et al. developed an analytical method for the determination of CYA in apples using liquid chromatography–tandem mass spectrometry (LC-MS/MS) following extraction with acetonitrile and ammonia. However, the method involves a single substrate and cannot be applied to the detection of residues in other substrates [14]. Therefore, studies about the detection methods and residue data of chloroisobromine cyanuric acid residues are lacking. To the best of our knowledge, no relevant studies have been established on pretreatment and monitoring methods or have evaluated the dietary risk assessment of chloroisobromine cyanuric acid in pepper and ginger.

The objectives of this study were to (1) develop a highly selective and sensitive analytical method for detecting low levels of CYA residues in pepper and ginger; (2) determine the residue levels of CYA in pepper and ginger through supervised field trials in China; and (3) conduct a dietary risk assessment of chloroisobromine cyanuric acid in pepper and ginger. The findings could provide essential data to support potential registration and a reference for residue analysis in other crops.

## 2. Materials and Methods

### 2.1. Reagents and Materials

The 50% chloroisobromine cyanuric acid soluble powder (SP) used in this study was provided by Cangzhou lanrun Biopharmaceutical Co., Ltd. (Cangzhou, Hebei Province, China). The analytical reference standard of CYA (99.5% purity) was purchased from CHEM SERVICE, Inc. The high-performance liquid chromatography (HPLC)-grade acetonitrile was purchased from Thermo Fisher Scientific Co. (Waltham, MA, USA). The octadecylsilane chemically bonded silica (C_18_, 40–60 μm), ethylenediamine-N-propyl silanized silica gel (precipitated silica adsorbent (PSA)), and graphitized carbon black (GCB, 120–400 mesh) were obtained from Agela technologies (Tianjin, China). Poly tetra fluoroethylene syringe filters (0.22 μm) were purchased from Chuding Technology Co., Ltd. (Shanghai, China). Additional analytical-grade reagents, such as anhydrous magnesium sulfate, were supplied by Sinopharm Chemical Reagent Co., Ltd. (Shanghai, China).

### 2.2. Standard Solution Preparation

A stock solution of CYA (1159.7 mg·L^−1^) and standard solutions were diluted with acetonitrile to prepare a series of appropriate solutions at appropriate concentrations. For the construction of calibration curves for quantitative analysis, matrix-matched standard solutions with concentrations of 0.002, 0.01, 0.02, 0.1, and 0.2 mg·L^−1^ were prepared by incorporating the standard solution into the blank matrix of pepper or ginger.

### 2.3. Field Trials and Sampling

The field trials for terminal residue tests on pepper and ginger were carried out in 12 and 4 representative regions of China in 2021, respectively. The dissipation tests for pepper were conducted in four provinces at the same time, which were designed following NY/T 788-2018 issued by the MARA [15]. 

At each ginger trial site, 50% chloroisobromine cyanuric acid SP was spread once on the soil surface before planting at the highest recommended dosage of 1500 g a.i.·ha^−1^. Since the edible part of ginger had not yet formed at the time of application, a dissipation study was not required according to NY/T 788-2018, and the terminal residue samples were sampled at the normal harvest time.

For pepper, the pesticide was sprayed three times at ten-day intervals at the early stage of disease, using the highest recommended dosage of 525 g a.i.·ha^−1^. For the pepper dissipation study, samples were collected at 2 h, 1, 2, 3, and 5 days after the last spraying. For the terminal residue study, samples were collected at 3 days and 5 days after the last spraying. Meanwhile, water was sprayed in the same manner on the control plot, which was separated by a buffer zone to avoid cross-contamination for the ginger and pepper.

### 2.4. Sample Extraction and Purification

The sample extraction and cleanup procedures were based on the QuEChERS method with minor modifications [16]: 4.0 g of ginger/pepper powder was precisely weighed and extracted with 20 mL acetonitrile in a centrifuge tube. The tube was shaken for 5 min at 2500 rpm, then centrifuged for 5 min at 4000 rpm. Next, 5 mL supernatant was added to a 10 mL tube containing 1.5 g anhydrous magnesium sulfate. The mixture was shaken for 5 min at 2500 rpm and centrifuged for 5 min at 4000 rpm. Thereafter, 3 mL supernatant was added to a 10 mL tube containing 3 mL distilled water and vortexed for 1 min. The resulting mixture was subjected to the clean-up procedure: 1.0 mL of mixture was transferred into a 2 mL tube including 20 mg of C_18_, 10 mg of PSA, and 10 mg of GCB. The mixture was vortexed for 30 s and centrifuged. Finally, the obtained solution was passed through a 0.22 μm syringe filter for LC-MS/MS analysis.

### 2.5. Instrument Conditions

The separation and quantification of CYA in pepper and ginger were conducted on an LCMS 8050 (Shimadzu 8050 Series, Kyoto, Japan) with an electrospray ionization interface. Chromatographic separation was performed on a Shim-pack Scepter Diol-HILIC-120 (1.9 μm, 2.1 × 100 mm) (Shimadzu). The flow rate was 0.2 mL/min. The column temperature was 40 °C. Mobile phase A was water and mobile phase B was acetonitrile. The injection volume was 5 μL. The mobile phase gradient elution is listed in Table 1.

The optimal MS parameters are presented in Table 2. The detailed MS source conditions were as follows: ionization mode: negative electrospray ionization (ESI) mode; monitoring method: multiple reaction monitoring; atomizing gas: N_2_, at a rate of 2 L/min; heating gas: N_2_, at a rate of 10 L/min; drying gas: air, at a rate of 10 L/min; ion source temperature: 300 °C; DL temperature: 250 °C; heat block temperature: 400 °C.

### 2.6. Analytical Method Validation

A recovery test was used to validate the sample preparation method. For the recovery assays, untreated pepper and ginger (blank control samples) were spiked with standard solutions. Five replicates of the spiked samples were prepared at three different analyte concentrations: 0.05, 1, and 4 mg·kg^−1^ for pepper, and 0.02, 1, and 4 mg·kg^−1^ for ginger. All samples were thoroughly mixed and incubated for 40 min before being extracted using the previous method. The accuracy and reproducibility of the method were assessed using relative standard deviation (RSD). In this study, the limit of quantification (LOQ) was defined as the lowest spiked concentration, which was 0.02 mg kg^−1^ for pepper and 0.05 mg·kg^−1^ for ginger.

### 2.7. Storage Stability Study

During the storage of experimental samples, the pesticide may degrade or undergo changes, potentially impacting the accuracy of the quantitative assay. According to NY/T 3094-2017 [17], a stability test is not necessary if samples are assayed within 30 days after harvest. All field samples of ginger were tested within 30 days after harvest, so no storage stability tests were conducted.

For pepper, the CYA standard solution was added into the blank matrix to achieve an initial concentration of 5 mg·kg^−1^. Subsequently, all samples were sealed and stored at temperatures below −18 °C. Sampling was conducted at intervals of 0 d, 98 d and 146 days. The samples were processed using the established method, and the degradation rate of CYA in pepper was calculated based on the formula in NY/T 3094-2017 [17].

### 2.8. Data Analysis

#### 2.8.1. Residue of Chloroisobromine Cyanuric Acid

Based on the residue definition outlined in the 2020 version of the list of Residues to be tested in pesticide registration residue test and plant-derived food dietary risk assessment, issued by the MARA, 2020, the terminal residues of chloroisobromine cyanuric acid were calculated using the following formula.

$$C_{Chloroisobromine\;cyanuric\;acid}=C_{CYA}\times 240.89÷129.07$$

where C_CYA_ is the detected concentrations (mg·kg^−1^);

240.89 is the molecular weight of Chloroisobromine cyanuric acid;

and 129.07 is the molecular weight of CYA.

In principle, the LOQ value is used for calculation when the detected concentration falls below the LOQ.

#### 2.8.2. Dissipation Curve and Half-Life

The dissipation curves in pepper were fitted using first-order kinetics, calculated as follows [18,19]:

C_t_ = C_0_ e^−kt^ (1)

t_1/2_ = ln 2/k (2)

where

C_0_ is the initial concentration (mg kg^−1^) at 2 h after application; 

C_t_ is the concentration (mg kg^−1^) of pesticide residues at time t (d);

k is the rate constant, which was used to measure the half-life (t_1/2_).

#### 2.8.3. Dietary Risk Assessment

The chronic dietary risk, expressed by risk probability, was calculated using the following formula [20].

NEDI=∑STMRi×Fi

$$Risk\;probability=\frac{NEDI}{ADI\times BW}\times 100\%$$

where

NEDI (mg) is the national estimated daily intake;

STMRi represents the supervised trial median residue of a pesticide’s active ingredient in a food commodity (mg kg^−1^); 

Fi is the consumption of a food commodity by the general population (kg·d^−1^);

ADI is the acceptable daily intake of CYA, which is 0.007 mg·kg^−1^ body weight.

BW is the average body weight of Chinese adults (i.e., 63 kg here). 

When calculating the NEDI, if a STMRi is not available, the corresponding MRL can be used instead [6,21]. 

Risk probability values of 100% or less indicate an acceptable level of risk. Conversely, when risk probability value exceeds 100%, the dietary risk posed by the pesticide active ingredient is unacceptable for the general population [18,19].

## 3. Results and Discussion

### 3.1. Optimization and Validation of the Analytical Methodology

#### 3.1.1. Optimization of the Method

One precursor ion and two generated product ions were chosen as identification ions. The optimized collision energy (CE) is shown in Table 2. Since ionization in electrospray mass spectrometry takes place in the state of liquid, the composition of the mobile phase affects not only the retention time and peak shape of the substance to be measured, but also the ionization efficiency of the mass spectrometry. In the preliminary experiments, the mobile phases, such as methanol and acetonitrile, purified water, 0.1% formic acid water, and 5 mmol/L ammonium acetate water solutions were compared and the results showed that CYA could not be ionized when either methanol or 0.1% formic acid water were used as the mobile phase. Precursor ions could only be obtained when the mobile phase A was pure water and B was acetonitrile in negative electrospray ionization (ESI) mode.

**Figure 1:**
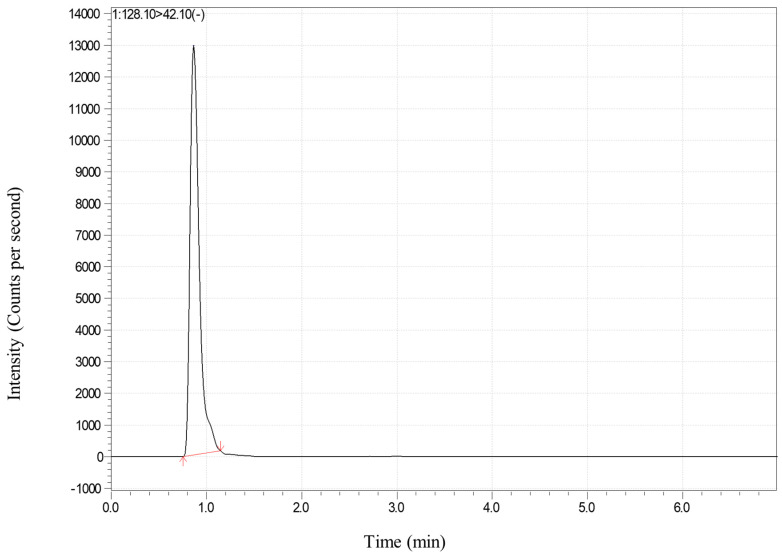
The LC−MS/MS chromatogram of CYA in ginger: matrix−matched standard solution at the spiked level of 0.5 mg kg^−1^.

**Figure 2:**
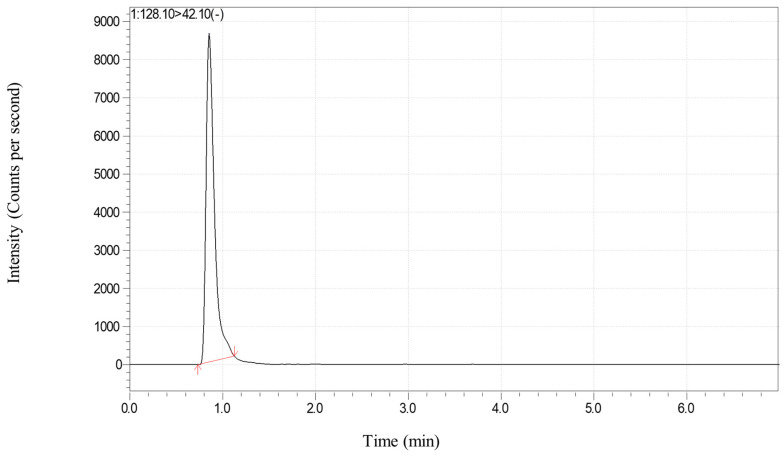
The LC−MS/MS chromatogram of CYA in pepper: matrix−matched standard solution at the spiked level of 0.5 mg kg^−1.^

Secondly, the ion source temperature has a great influence on the ionization efficiency of CYA. The maximum response value could be obtained when the ion source temperature was in the range of 300~400 °C and the atomization gas was set to 2 L/min; the response value at 200 °C was only one quarter of the maximum value; and if the temperature was too high, the depolymerization reaction of CYA could have occurred easily, which could affect the sensitivity and stability of the method. Therefore, the ion source temperature of 300 °C was chosen for the method. In addition, the total ion chromatograms of the matrix-matched standard solution are shown in Figure 1 and Figure 2. 

The matrix has a big impact on the detection of CYA. It was found that other acids in the sample had a great influence on the analysis of CYA, and it could not be detected by mass spectrometry without the step of removing the other acids in the sample, so deacidification using PSA is both simple and feasible. However, CYA can be absorbed by PSA because it is also an acid, but a less acidic one. It was found that adding distilled water to the extraction solution solved this problem. The adsorption results of PSA on CYA are shown in Table 3. GCB and C_18_ had no adsorption effect on CYA, and they could remove the pigments and nonpolar interfering components in the extract, which reduced the effect of the matrix and increased the sensitivity of the mass spectrometry to the analytes to be measured. Ultimately, 20 mg of C_18_, 10 mg of PSA, and 10 mg of GCB were used.

#### 3.1.2. Linearity and Precision

The matrix effect can significantly interfere with the analytical process and compromise the accuracy of the results. To address this issue, matrix-matched calibration curves were employed to compensate for matrix effects. The linearity was assessed by fortifying blank samples at six concentration levels and good linearity was observed with R^2^ > 0.99 (Table 4). As shown in Table 5, the average recoveries of ginger ranged from 91% to 96% and the RSD ranged from 2.9% to 11.2%, while the average recoveries of the pepper samples ranged from 84% to 89% and the RSD ranged from 2.8% to 12.9%, which were in accordance with the standards described in the SANTE guidelines [22] and NY/T 788-2018. The LOQ was the lowest addition level of the analysis method at 0.02 mg kg^−1^ and 0.05 mg kg^−1^ for ginger and pepper, respectively, which were lower than the MRL of CYA in pepper. The value meets the criteria of the Chinese National Standard NY/T 788-2018, which stipulates that LOQs should be below 0.05 mg/kg or no higher than the MRL [15].

All these results indicate that our methods can meet the requirements for analyzing CYA in ginger and pepper. 

### 3.2. Storage Stability Study

Following the requirements of NY/T 3094-2017 [17], the concentration of pesticide residues in storage stability samples should be at least 10 times the LOQ. For pepper, where the MRL of CYA was 5 mg kg^−1^, the level for the storage stability test was set as 5 mg kg^−1^. 

The results indicated that the average degradation rate of CYA was 8.7% at 98 days and 26.1% at 146 days when stored at temperatures below −18 °C (Table 6). Based on NY/T 3094-2017, a degradation rate of less than 30% of pesticides during storage indicates stable storage conditions. Therefore, the storage stability period of CYA in pepper was determined to be at least 146 days. In this study, all pepper samples were collected and analyzed within the storage stability period.

### 3.3. Dissipation Kinetics in Pepper

The dissipation of a pesticide after application is an important indicator for assessing its residue behavior [23,24]. Chloroisobromine cyanuric acid residues in pepper samples were determined using a matrix-based external standard method. None of the blank control pepper samples had detectable residues of chloroisobromine cyanuric acid. As the data suggest (Table 7), the half-lives of chloroisobromine cyanuric acid and were determined to be 3.5 days in peppers from Shandong Province and 3.1 days in peppers from Anhui Province. In contrast, the half-lives were 7.4 days in Guizhou Province and 8.2 days in Guangxi Province peppers. The dissipation behavior of chloroisobromine cyanuric acid in peppers followed the first-order kinetics model, with a good correlation coefficient (R^2^) value ranging from 0.8145 to 0.9851.

The dissipation rate of a pesticide depends on the plant matrix and other environmental factors such as humidity, temperature, sunlight, and soil conditions [25,26,27]. The differences in cultivation may be a key factor influencing the dissipation rate of chloroisobromine cyanuric acid in pepper.

### 3.4. Terminal Residue Levels

The terminal residue levels of chloroisobromine cyanuric acid in ginger from 4 representative cultivation regions and pepper from 12 regions were detected and are listed in Table 8. Residual levels should be expressed as <LOQ when they are below the LOQ (MARA, 2018). 

The residue of chloroisobromine cyanuric acid in ginger was in the range of <0.037–0.054 mg kg^−1^ when the ginger samples were collected at normal harvest time.

In pepper, the residue contents of chloroisobromine cyanuric acid on days 3 and 5 after the last treatment were 0.37–1.6 mg kg^−1^ and 0.34–1.5 mg kg^−1^, respectively.

All the terminal residues were less than the MRL in pepper (5 mg kg^−1^) established by the Chinese government (GB 2763-2021, issued by the MARA). The recommended PHI for ginger is normal harvest time and for pepper it is 3 days [6].

### 3.5. Dietary Risk Assessment

The dietary intake risk assessment of chloroisobromine cyanuric acid was conducted by comparing the NEDI with the ADI, based on the terminal residual data from field experiments. 

As a domestically produced fungicide independently developed by China in the 1980s, chloroisobromine cyanuric acid has not yet been registered in foreign countries, and there are no relevant MRL standards in developed countries (i.e., USA, Japan, Australia, and the Republic of Korea) or international organizations (i.e., Codex Alimentarius Commission/CAC and the European Union). Thus, as shown in Table 9, each crop was classified into a food commodity category and the corresponding MRLs were referenced from GB 2763-2021. 

Subsequently, reference MRLs for each food commodity category were calculated with the highest values selected as the STMRi for that category. However, for the soy sauce category (which includes ginger) and dark-colored vegetables (which include pepper), the STMR was used (Table 7). The NEDI values for chloroisobromine cyanuric acid were calculated to be 0.16740 mg, with risk possibilities of 37.96%. The RQ value was much lower than 100%, indicating that the application of 50% chloroisobromine cyanuric acid SP on ginger and pepper under Good Agricultural Practices (GAP) conditions in China does not pose an unacceptable dietary risk to the Chinese population.

## 4. Conclusions

This study established a QuEChERS-LC-MS/MS method for the determination of chloroisobromine cyanuric acid residues in pepper and ginger and elucidated the residue fates of chloroisobromine cyanuric acid in pepper and ginger for the first time. The half-life of CYA in pepper was 3.1~8.2 d. The final residue level of CYA in ginger was less than 0.054 mg·kg^−1^. Its final residue level in pepper was less than 1.6 mg·kg^−1^, which was lower than the MRL of 5 mg·kg^−1^ in China. The probability of chronic dietary risk of chloroisobromine cyanuric acid for the general population was 37.96%, within the acceptable level, indicating no significant health risk to the Chinese population. 

## Figures and Tables

**Table 1:** Gradient elution procedure.

Time (Min)	Phase A (%)	Phase B (%)	Flow Rate (mL/Min)
0.01	20	80	0.3
1.50	20	80	0.3
1.51	60	40	0.3
4.00	60	40	0.3
4.01	20	80	0.3
7.00	Stop		0.3

Note: Mobile phase A was water and mobile phase B was acetonitrile.

**Table 2:** LC-MS/MS parameters.

	ESI	Retention Time (Min)	Precursor Ion (*m*/*z*)	Product Ion (*m*/*z*)	Collision Energy (eV)	Dwell Time (ms)
CYA	(-)	0.83	128.1	42.1 *	13	70
				85.0	11	

Note: * ions are used for quantification.

**Table 3:** Adsorption of CYA by PSA.

	Acetonitrile	Acetonitrile:Water = 8:2	Acetonitrile:Water = 5:5	Acetonitrile:Water = 2:8	Water
Adsorption ratio (%)	82.5	41.1	10.8	4.37	0

**Table 4:** Calibration curves and limit of quantification (LOQ) of CYA in ginger and pepper.

Compound	Matrix	Matrix-Matched Standard Calibration Curve	Range (mg L^−1^)	r	LOQ (mg kg^−1^)
CYA	Ginger	y = 181961x + 25.4342	0.001–0.5	0.9967	0.02
	Pepper	y = 415883x + 476.279	0.002–0.2	0.9996	0.05

**Table 5:** Average recoveries of CYA in ginger and pepper (n = 5).

Matrix	Spiked Concentration (mg kg^−1^)	Recovery (%)						RSD (%)
		1	2	3	4	5	Average	
Ginger	0.02	110	100	85	95	85	95	11.2
	1	99	98	93	97	93	96	2.9
	5	94	90	88	94	90	91	3.0
Pepper	0.05	90	66	82	86	94	84	12.9
	1	93	87	88	88	91	89	2.8
	5	86	88	92	86	86	88	3.0

**Table 6:** Stability of CYA in pepper following frozen storage.

Spiked Level (mg kg^−1^)	Storage Interval (days)	Residues (mg kg^−1^)	Degradation Rates (%)	Freshly Fortified Controls (%)
5	0	4.6	/	89
	98	4.2	8.7	89
	146	3.4	26.1	83

**Table 7:** Dissipation dynamics parameters of chloroisobromine cyanuric acid in pepper.

Matrix	Locations	Dynamic Equations	Correlation Coefficient (R^2^)	Half-Life (*t*_1/2_, Day)
Pepper	Shandong (greenhouse cultivation)	C = 0.9744e^−0.200t^	0.9851	3.5
	Anhui (greenhouse cultivation)	C = 1.2091e^−0.223t^	0.9113	3.1
	Guizhou (open-air cultivation)	C = 1.1977e^−0.094t^	0.9735	7.4
	Guangxi (open-air cultivation)	C = 2.1943e^−0.085t^	0.8145	8.2

**Table 8:** Terminal residue levels of chloroisobromine cyanuric acid in ginger and pepper.

Matrix	PHI ^a^ (d)	Residues (mg kg^−1^)	STMR ^b^ (mg kg^−1^)	HR ^c^ (mg kg^−1^)
Ginger	harvest	<0.037 (5) ^d^, 0.045 (2), 0.054	<0.037	0.054
Pepper	3	0.37 (2), 0.45, 0.47, 0.50, 0.52, 0.54, 0.62, 0.63, 0.65, 0.82, 0.90 (2), 0.95, 0.97, 1.0, 1.2, 1.3 (2), 1.4, 1.5 (3), 1.6	0.90	1.6
	5	0.34, 0.35, 0.37 (3), 0.43, 0.45, 0.47, 0.50, 0.58 (2), 0.65, 0.75 (2), 0.78, 0.82, 0.90, 0.95, 1.1, 1.2 (3), 1.5 (2)	0.70	1.5

^a^ PHI, pre-harvest interval days. ^b^ STMR, supervised trial median residue. ^c^ HR, highest residue. ^d^ <0.037 (5), the terminal residual value below LOQ is expressed as <0.037, and the number in bracket indicates the number of residual values. The data underlined are used to calculate the STMR.

**Table 9:** Dietary intake risk assessment of chloroisobromine cyanuric acid and the corresponding MRLs registered by China according to GB 2763-2021.

Registration Crop	Food Category	Dietary Intake (mg kg BW^−1^)	Reference MRL/STMR (mg kg^−1^)	NEDI (mg)	ADI × BW (mg)	Risk Possibility (%)
Rice	Rice and processed products	0.2399	0.2	0.04798	0.007 × 63	37.96
Pepper, tomato	Dark-colored vegetables	0.0915	0.90 (STMR)	0.08235		
Chinese cabbage, cucumber	Light-colored vegetables	0.1837	0.2	0.036740		
Ginger	Soy sauce	0.009	0.037 (STMR)	0.000333		
Total		1.0286	/	0.16740	0.441	

## Data Availability

The original contributions presented in the study are included in the article; further inquiries can be directed to the corresponding author.
